# A comparable efficacy and safety between intracardiac echocardiography and transesophageal echocardiography for percutaneous left atrial appendage occlusion

**DOI:** 10.3389/fcvm.2023.1194771

**Published:** 2023-05-24

**Authors:** Zhi-Yuan Zhang, Feng Li, Jie Zhang, Lei Zhang, Huan-Huan Liu, Ning Zhao, Fan Yang, Qi Kong, Yi-Ting Zhou, Ling-Ling Qian, Ru-Xing Wang

**Affiliations:** Department of Cardiology, Wuxi People’s Hospital Affiliated to Nanjing Medical University, Wuxi, China

**Keywords:** atrial fibrillation, intracardiac echocardiography, transesophageal echocardiography, left atrial appendage closure, implantable devices, cardiac mapping

## Abstract

**Background:**

Accumulated clinical studies utilized intracardiac echocardiography (ICE) to guide percutaneous left atrial appendage occlusion (LAAO). However, its procedural success and safety compared to traditional transesophageal echocardiography (TEE) remained elusive. Therefore, we performed a meta-analysis to compare efficacy and safety of ICE and TEE for LAAO.

**Methods:**

We screened studies from four online databases (including the Cochrane Library, Embase, PubMed, and Web of Science) from their inception to 1 December 2022. We used a random or fixed-effect model to synthesize the clinical outcomes and conducted a subgroup analysis to identify the potential confounding factors.

**Results:**

A total of twenty eligible studies with 3,610 atrial fibrillation (AF) patients (1,564 patients for ICE and 2,046 patients for TEE) were enrolled. Compared with TEE group, there was no significant difference in procedural success rate [risk ratio (RR) = 1.01; *P* = 0.171], total procedural time [weighted mean difference (WMD) = −5.58; *P* = 0.292], contrast volume (WMD = −2.61; *P = *0.595), fluoroscopic time (WMD = −0.34; *P* = 0.705; *I*^2^ = 82.80%), procedural complications (RR = 0.82; *P = *0.261), and long-term adverse events (RR = 0.86; *P* = 0.329) in the ICE group. Subgroup analysis revealed that ICE group might be associated with the reduction of contrast use and fluoroscopic time in the hypertension proportion <90 subgroup, with lower total procedure time, contrast volume, and the fluoroscopic time in device type subgroup with multi-seal mechanism, and with the lower contrast use in paroxysmal AF (PAF) proportion ≤50 subgroup. Whereas, ICE group might increase the total procedure time in PAF proportion >50 subgroup and contrast use in multi-center subgroup, respectively.

**Conclusion:**

Our study suggests that ICE may have comparable efficacy and safety compared to TEE for LAAO.

## 1. Introduction

Atrial fibrillation (AF) is the most common persistent atrial arrhythmia worldwide, with a prevalence estimated to be between 2% and 4% in adults. An expected 2.3-fold increase in prevalence is anticipated due to extended life expectancy in the general population and increased detection of undiagnosed AF (1). Cardioembolic stroke is the most concerning complication of AF, as abnormal blood flow in the left atrium increases the likelihood of thrombus rupture from the left atrial appendage (LAA), subsequently leading to thromboembolisms in the peripheral and cerebral arteries (2).

The primary prevention strategy of thromboembolism for AF is the use of oral anticoagulants (OACs). However, challenge remains due to the limitation of adherence and bleeding risk for safety and efficacy of OACs. Since most thrombus in nonvalvular AF originates from the LAA, left atrial appendage occlusion (LAAO) is an emerging alternative for OACs. Transesophageal echocardiography (TEE) is the standard imaging modality to guide LAAO and is the most widely used imaging modality. However, it has some significant limitations, including increased pain with local or conscious anesthesia, prolonged procedure time and hospitalization burden with general anesthesia, aggravated risk of possible esophageal injury under “one-stop” ablation, and high dependence on a dedicated echocardiography operator.

Recently, an expert consensus suggested that intracardiac echocardiography (ICE) might be considered as an alternative imaging modality to guide LAAO, especially with the progress of the “one-stop” ablation therapy for AF (3). However, studies comparing TEE with ICE for LAAO were limited, leading to the related outcomes (e.g., efficacy and safety outcomes) remaining elusive. Therefore, we evaluated the clinical outcomes of TEE and ICE guidance for LAAO to further assess the safety and efficacy outcomes between two imaging modalities.

## 2. Methods

### 2.1. Study design

This systematic review was carried on according to the PRISMA guidelines. The registered protocol is displayed in the PROSPERO database (CRD42022368692).

### 2.2. Search strategy

Two independent reviewers (ZYZ and FL) conducted comprehensive searches of four online databases (Cochrane Library, Embase, PubMed and Web of Science) from inception to 1 December 2022. Search keywords were “ICE”, “Intracardiac echocardiography”, “TEE “, “transesophageal echocardiography”, “atrial fibrillation”, “left atrial appendage closure”, “LAAC”, “left atrial appendage occlusion”, and “LAAO”. Clinical studies related to the outcomes of ICE or outcomes comparing TEE vs. ICE for LAAO were included. Reference lists of review articles were hand searched, and eligible articles were searched for potential publications not previously identified.

### 2.3. Search design

Two reviewers (ZYZ and JZ) independently searched the literature and screen the titles, abstracts, and full texts to select all relevant studies that met the inclusion criteria. A study would be included if the following criteria were met: (1) randomized controlled trials and cohort, observational studies, and single-arm studies; (2) studies comparing clinical outcomes comparing TEE vs. ICE for endocardial LAAO, including efficacy outcome (e.g., procedural success) and safety outcomes (e.g., short-term complications and long-term complications); (3) studies with full text published in peer-reviewed journals; and studies containing the most data for multiple publications of the same study. Case reports, editorial, review articles, studies without original data letters and studies reporting clinical outcomes with hybrid LAAO procedures were excluded. Meanwhile, a third reviewer (R.X.W) resolved any disagreements about eligibility.

### 2.4. Data extraction and quality assessment

Data from eligible studies included in the analysis were extracted by two independent researchers (ZYZ and FL), and any potential disagreements were resolved by a third researcher (RXW). The extracted data mainly included: title, first author, publication year, study design, sample size, follow-up time, LAAO device, pre-procedure imaging and ICE location. Meanwhile, we also extracted relevant clinical outcomes, including: acute procedural success, total procedural time, fluoroscopic time, contrast volume, short-term complications, and long-term complications.

Two independent researchers (ZYZ and JZ) evaluated study quality by two appraisal tools. The Newcastle-Ottawa Quality Assessment Scale (NOS) was used to evaluated the two-arm observation (4). The Institute of Health Economics checklist was used for the single-arm study (5). Any disagreements were discussed and resolved by consulting a third researcher (RXW).

### 2.5. Statistical analysis

Stata version 16.0 was used for statistical analyses. Continuous variables were displayed as means ± SD, and categorical variables were presented as frequencies and percentages. For observational studies with two arms, we calculated the relative risk (RR) and corresponding 95% confidence intervals (CI) for each outcome. For single-arm analysis, we calculated the incidence of events (number of events divided by number of patients) and 95% confidence intervals. *P *< 0.05 was considered statistically significant.

Meanwhile, chi-square tests and *I*-squared (*I*^2^) were used to quantify and assess statistical heterogeneity among studies. If the *I*^2^ value was more than 50% and/or *P* < 0.05 for the chi-squared test, we considered the between-study heterogeneity to be significant, and we would adopt a random-effect model. Otherwise, we would adopt fixed-effect model. Sensitivity analysis was performed by sequentially omitting one study at a time to assess the effect of a single study on the overall risk, and potential publication bias was also evaluated *via* Egger's test.

In addition, subgroup analysis was conducted to screen potential determinants of LAAO outcomes between ICE and TEE groups. According to the characteristics of eligible studies, a total of eight subgroup factors were identified, including study design, age cutoff, ICE group sample size, AF type, male proportion, hypertension proportion, device types, and duration of follow up. If the study design included more than one center, it was defined as a multicenter subgroup; otherwise, it was defined as a single-center subgroup. According to age cutoff values of 75, two subgroups were divided, including ≥75 years subgroup and <75 years subgroups. If over 50% of patients had paroxysmal AF (PAF), they were classified as ≥50% PAF subgroup, otherwise they were classified as <50% PAF subgroup. According to the proportion of the male, they were divided into ≥70% subgroups and <70% subgroups. Similarly, the proportion of hypertension with ≥90% subgroup and <90 subgroup, respectively, also was defined. According to the sealing position, the existing sealers could be roughly divided into plug type and disc type. Plug type sealers, also known as single sealers, included Watchman, Plaato, and Lefort. Disc sealer was also called dual sealer, including ACP, Lambre, Lacbes, and Leftear. If the LAAO devices included only dual-seal mechanism devices, it was assigned to dual-seal mechanism subgroup, and if the LAAO devices included only single-seal mechanism devices, it was assigned to the single-seal mechanism subgroup. In addition, studies using both dual-seal mechanism devices and single-seal mechanism devices were divided into muti-seal mechanism subgroup. Follow-up time was divided into two subgroups (≥12 months and <12 months).

## 3. Results

### 3.1. Study selection and quality assessment

**Figure 1:**
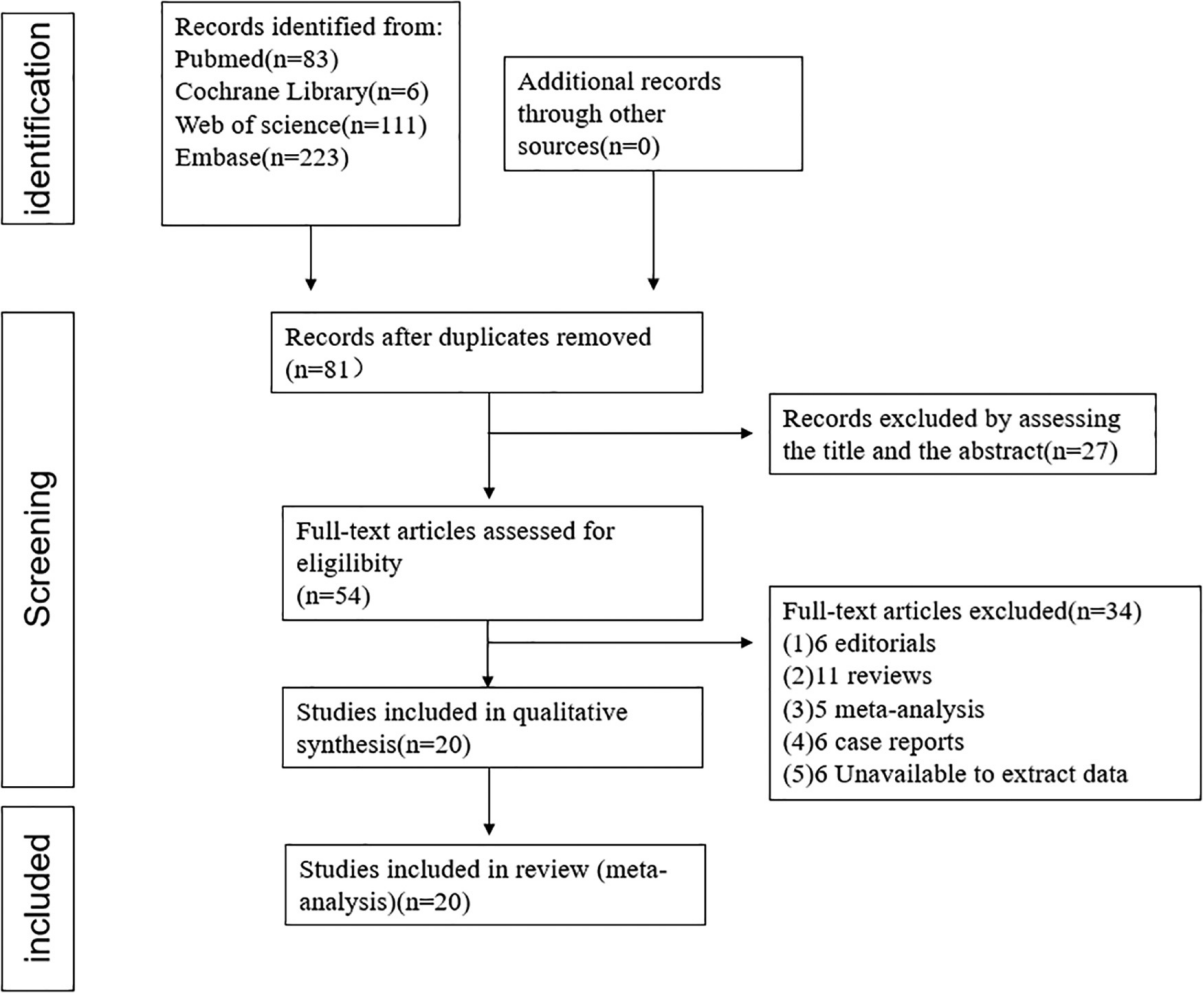
The flowchart of the study selection.

This meta-analysis included 20 studies with a total of 3,610 AF patients (1,564 patients for ICE and 2,046 patients for TEE) consisting of 10 observational two-arm studies (965 ICE patients and 2,046 TEE patients) (6–15) and 10 single-arm studies (599 ICE patients) (16–25). The selection flowchart was displayed in Figure 1. The average age of the patients included in the studies ranged from 71.3 to 80.3 years. Among the included clinical studies, the mean CHA_2_DS_2_-VASc score and HAS-BLED score ranged from 3.9 to 5.3 and 2.4 to 4.4. Eleven studies included Watchman or Watchman FLX (6, 8, 9, 13, 16, 17, 19–23), six included the ACP or Amulet device (10, 11, 14, 18, 24, 25) and three studies included both (7, 12, 14). The baseline characteristics and procedure-related indexes of the eligible studies were presented in Table 1. In this meta-analysis, all two-arm studies had a moderate-to-high quality (Supplementary Table S1). Ten single-arm studies all had a score higher than fifteen (Supplementary Table S1).

**Table 1:** Baseline characteristics and procedure-related indices of eligible studies.

First author	Year	Study design	Follow-up (Months)	ICE sample size		Gender (male%)		Age		Hypertension (%)		PAF (%)		LAAO device	
				ICE Group	TEE Group	ICE Group	TEE Group	ICE Group	TEE Group	ICE Group	TEE Group	ICE Group	TEE Group	ICE Group	TEE Group
Gianni	2021	Single-center	2	122	68	66	60	72 ± 8	75 ± 9	NA	NA	NA	NA	Watchman FLX	
Pommier	2021	Single-center	1	175	49	70	73	76 ± 8	75 ± 7	91	96	29	23	Watchman/ACP	
Alkhouli	2020	Single-center	1.5	90	196	62.2	55.6	75.7 ± 8.0	75.2 ± 7.8	92.2	87.2	NA	NA	Watchman	
Hemam	2019	Multi-center	4	53	51	62.3	60.8	77 ± 10	76 ± 7	81	90	NA	NA	Watchman	
Nielsen-Kudsk	2019	Multi-center	12	130	955	60	65	75 ± 8	75 ± 9	NA	NA	NA	NA	Amulet	
Berti	2018	Multi-center	15	187	417	66	65	76 ± 8	74 ± 7	NA	NA	32	36	ACP/Amulet	
Kim	2018	Multi-center	25.6	41	103	58.5	49.5	71.4 ± 9.3	72.3 ± 9.2	90.2	83.5	34.1	27.2	Watchman/ACP/Amulet	
Frangieh	2017	Single-center	0	32	44	81	57	74.6 ± 9.3	80.3 ± 7.7	84	86	69	54	Watchman	
Korsholm2	2017	Single-center	1.7	109	107	62	74	73.0 ± 7.8	73.0 ± 9.7	83	80	48	42	ACP/Amulet	
Reis	2018	Single-center	23	26	56	53		74 ± 8		86.6		30.5		Watchman/ACP/Amulet	
Dallan	2022	Single-center	1.5	136		55.1		76 ± 8.4		85.3		53.7		Watchman FLX	
Turagam1	2022	Single-center	12	15		33		71.3 ± 10.8						Watchman	
Chen	2022	Single-center	12	56		57.1		69.4 ± 7.5		69.6		41.1		LAmbre	
Turagam2	2021	Single-center	1.5	30		53		75.4 ± 8.4		90		NA		Watchman	
Filby	2021	Single-center	1.5	71		54.9		76 ± 8.8		81.7		59.2		Watchman/Watchman FLX	
Korsholm1	2020	Single-center	1.7	92		25		73.3 ± 8.5		76		51		Watchman FLX	
Khalili	2019	Single-center	0	15		73		75.6 ± 10		NA		13.3		Watchman	
Matsuo	2016	Single-center	1.5	27		40.7		77.0 ± 8.5		88.9		14.8		Watchman	
Masson	2015	Single-center	2	37		67.6		74.7 ± 8.2		94.6		51.3		ACP	
Berti	2014	Multi-center	0	121		57		77 ± 7.6		89.3		20.7		ACP/Amulet	

**Table:** 

First author	Pre-procedure imaging		CHA_2_DS_2_-VASc		HAS-BLED		Prior stroke (%)		Congestive heart failure (%)		History of CAD (%)		Diabetes mellitus (%)		ICE location	
	ICE Group	TEE Group	ICE Group	TEE Group	ICE Group	TEE Group	ICE Group	TEE Group	ICE Group	TEE Group	ICE Group	TEE Group	ICE Group	TEE Group	ICE Group	TEE Group
Gianni	CT/TEE		4.1 ± 1.4	4.3 ± 1.3	2.7 ± 1.3	2.7 ± 1.2	NA	NA	NA	NA	NA	NA	NA	NA	100%LA	
Pommier	CT		4.2 ± 1.38	4.5 ± 1.49	4.07 ± 0.99	3.93 ± 1.02	70	64	17	17	29	28	34	21	100%LA	
Alkhouli	CT/TEE		4.7 ± 1.4	4.8 ± 1.6	2.8 ± 1.2	2.9 ± 1.1	36.5	42.9	56.7	48.5	52.2	51	33.3	43.9	100%LA	
Hemam	NA		4.5 ± 1.8	4.5 ± 1.6	NA	NA	42	33	19	25	NA	NA	34	29	100%LA	
Nielsen-Kudsk	CT and TEE		4.1 ± 1.6	4.2 ± 1.6	3.3 ± 1.1	3.2 ± 0.9	42	25	NA		NA	NA	NA	NA	100%LA	
Berti	TEE	TEE	4.27 ± 1.40	4.25 ± 1.40	3.25 ± 1.00	3.15 ± 1.10	NA	NA	NA	NA	NA	NA	NA	NA	100%LA	
Kim	TEE	TEE	4.3 ± 1.4	4.3 ± 1.4	3.0 ± 1.5	3.1 ± 1.4	48.8	42.7	43.9	39.8			26.8	25.2	100%LSPV	
Frangieh	NA		4.3 ± 5.2	4 ± 1.5	3.6 ± 1.4	3.4 ± 0.8	28	21	NA	NA	63	61	36	44	100%LA	
Korsholm2	CT	CT	4.1 ± 1.6	4.4 ± 1.6	4.1 ± 0.9	4.1 ± 1.1	46	55	15	20	NA	NA	21	22	100%LA	
Reis	TEE	TEE	4.7 ± 1.4		3.3 ± 1.0		41.5		NA		22		86.6		100%LA	
Dallan	CT		4.4 ± 1.3		NA		NA		22.1		33.1		31.6		100%LA	
Turagam1	TEE		4.1 ± 1.7		3.4 ± 1.4		NA		NA		NA		NA		100%LA	
Chen	NA		4.0 ± 1.5		2.6 ± 0.8		46.4		41.1		19.6		28.6		100%LA	
Turagam2	NA		4.6 ± 1.6		3.4 ± 1.1		43.3		30				13.3		100%LA	
Filby	CT		4.2 ± 1.4		3.6 ± 1.0		21.1		22.5		49.3		28.2		100%LA	
Korsholm1	CT		3.9 ± 1.7		2.4 ± 1.0		42		16				26		100%LA	
Khalili	TEE		4.6 ± 1				NA		NA				NA		100%RA	
Matsuo	TEE		5.3 ± 1.6		4.4 ± 1.1		37		55.6				44.4		100%LA	
Masson	NA		4.5 ± 1.3		4.2 ± 0.7		40.5		NA				45.9		100%LA	
Berti	TEE		4.4 ± 1.3		3.3 ± 1.0		31.4		13.2				24		38%LA;62%RA	

Note: AF, atrial fibrillation; PAF, paroxysmal atrial fibrillation; LAAO, left atrial appendage closure; CAD, coronary artery disease; ICE, intracardiac echocardiography; TEE, transesophageal echocardiography; LA, left atria; RA, right atria.

### 3.2. Primary outcome

#### 3.2.1. Procedural success rate

**Figure 2:**
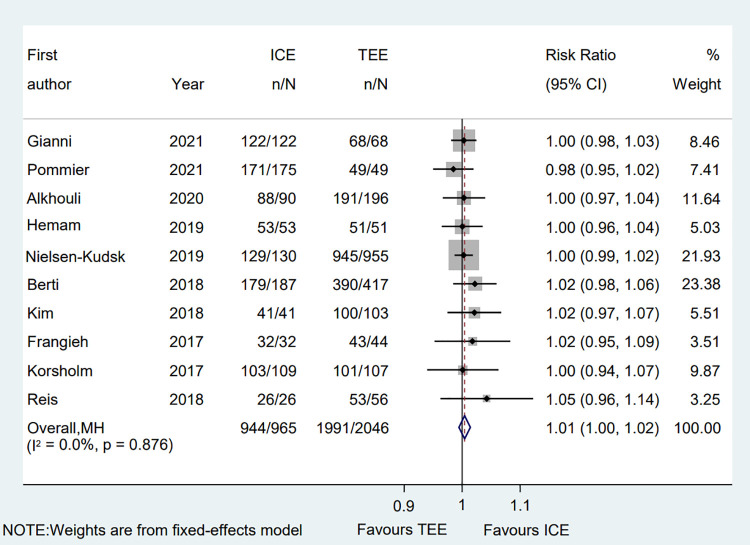
Forest plot of the procedural success between ICE and TEE groups. Comparison of the rates of the procedural success between ICE and TEE groups. ICE, intracardiac echocardiography; TEE, transesophageal echocardiography; RR, risk ratio; CI, confidence interval.

**Figure 3:**
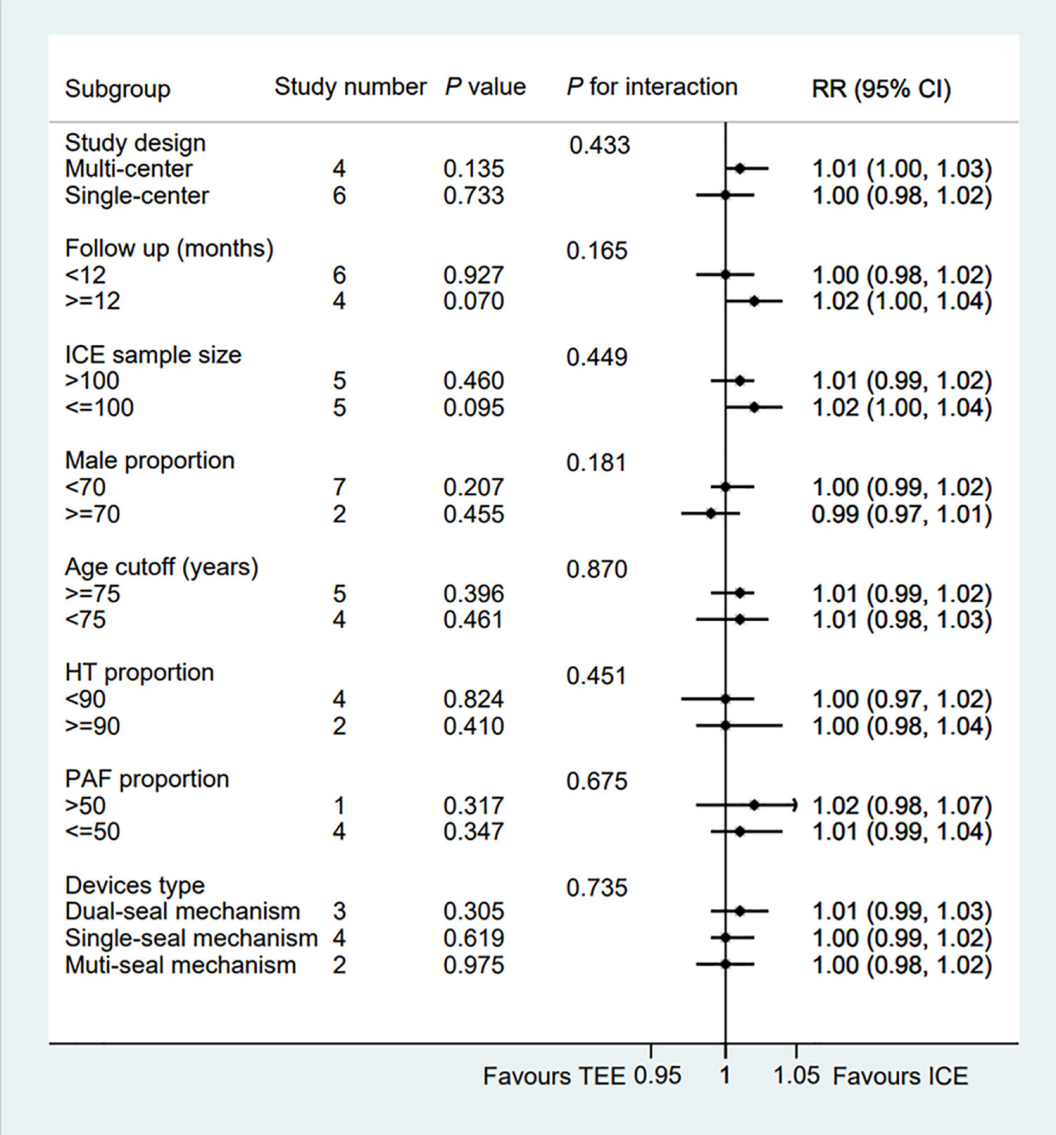
Forest plot of subgroup analysis of the procedural success between ICE and TEE groups. Subgroup analysis of the rates of the procedural success between ICE and TEE groups. ICE, intracardiac echocardiography; TEE, transesophageal echocardiography; RR, risk ratio; CI, confidence interval.

All eligible two-arms studies reported the acute procedural success data and there was no significant difference in procedural success rate (RR = 1.01; 95% CI: 1.00, 1.02; *P* = 0.171; *I*^2^ = 0.00%) between two groups (Figure 2) (6–15). Our result was consistent with those of several other meta-analyses (26–28). Subgroup analysis was performed with a total of eight subgroup factors for the acute procedural success of LAAO, and the results are displayed in Figure 3. There was no significant difference between TEE group and ICE group in the study design subgroup, follow-up subgroup, ICE sample size subgroup, male proportion subgroup, age cutoff subgroup, hypertension proportion subgroup, PAF proportion subgroup, and device types subgroup, suggesting that all subgroup results were consistent with the pooled result.

We also performed a sensitivity analysis and the results showed no significant change, ranging from 1.00 (95% CI: 0.99, 1.02) to 1.01 (95% CI: 1.00, 1.03), in the overall combined proportion, suggesting that there was no single study in the domination of the combined proportion and heterogeneity. Moreover, no publication bias was presented in Egger's test (*P* = 0.208).

#### 3.2.2. Pooled rate of procedural success in ICE group

**Figure 4:**
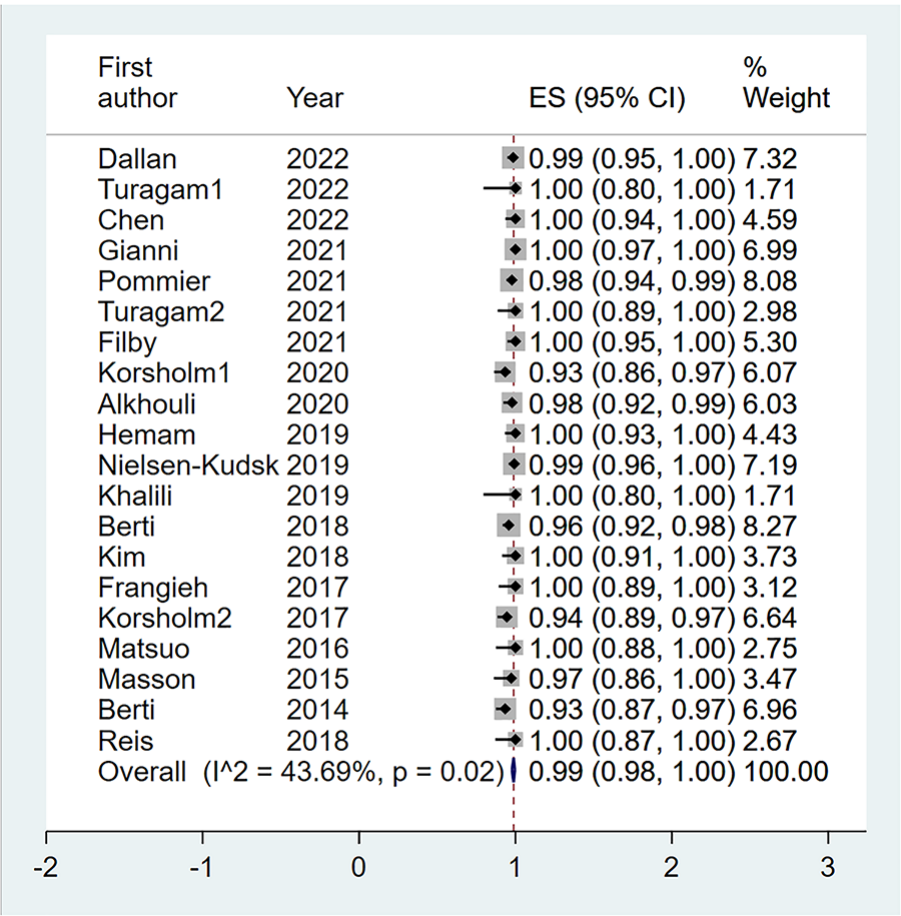
Forest plot of the pooled rate of the procedural success in ICE groups. The line of equity refers to the pooled result of eligible studies in the forest plots. ICE, intracardiac echocardiography; TEE, transesophageal echocardiography; ES, effect size; CI, confidence interval.

A total of 20 eligible studies (1,564 patients undergoing LAAO with ICE procedural guidance) reported the rate of procedural success in ICE Group (6–15). The pooled rate of procedural success was 0.99 (95% CI: 0.98, 1.00; *P* = 0.02; *I*^2^ = 43.69%) with the random-effect model (Figure 4). Meanwhile we performed a subgroup analysis with eight subgroup factors for procedural success in ICE group, and the results are shown in Table 2. Overall, the pooled rate of procedural success in ICE Group does not differ significantly between subgroups.

**Table 2:** Subgroup analysis of the rate of procedural success in ICE group.

Subgroup factors	Numbers of study	Pooled incidence	95% CI	*I*^2^ (%)	*P* for interaction
Study design					0.670
Multi-centered	5	0.99	(0.98, 1.0)	31.86	
Single-centered	15	0.98	(0.95, 1.0)	66.08	
ICE Sample size					0.280
>100	7	0.98	(0.95, 0.99)	68.51	
≤100	13	0.99	(0.98, 1.0)	4.01	
Male proportion					0.990
<70	16	0.99	(0.97, 1.0)	54.17	
≥70	3	0.99	(0.97, 1.0)	–	
Age cutoff					0.890
≥75	11	0.99	(0.97, 1.0)	35.33	
<75	8	0.99	(0.96, 1.0)	60.83	
HT proportion					0.720
<90	10	0.98	(0.96, 1.0)	57.21	
≥90	4	0.99	(0.97, 1.0)	0	
PAF proportion					0.580
>50	5	0.98	(0.96, 1.0)	52.71	
≤50	8	0.98	(0.96, 0.99)	42.03	
Devices type					0.230
Dual-seal mechanism	6	0.94	(0.94, 0.99)	57.64	
Single-seal mechanism	11	0.98	(0.98, 1.0)	26.89	
Muti-seal mechanism	2	0.96	(0.96, 1.0)	–	

ICE, intracardiac echocardiography; TEE, transesophageal echocardiography; CI, confidence interval.

Also, sensitivity analysis showed that no significant change was detected in the overall combined proportion, ranging from 0.98 (95% CI: 0.97, 0.99) to 0.99 (95% CI: 0.98, 1.00), indicating that no single study dominated the combined proportion and heterogeneity. Moreover, Egger's test was performed and result showed no publication bias (*P* = 0.068), which indicated that the results were robust.

### 3.3. Secondary outcome

#### 3.3.1. Total procedure time

**Figure 5:**
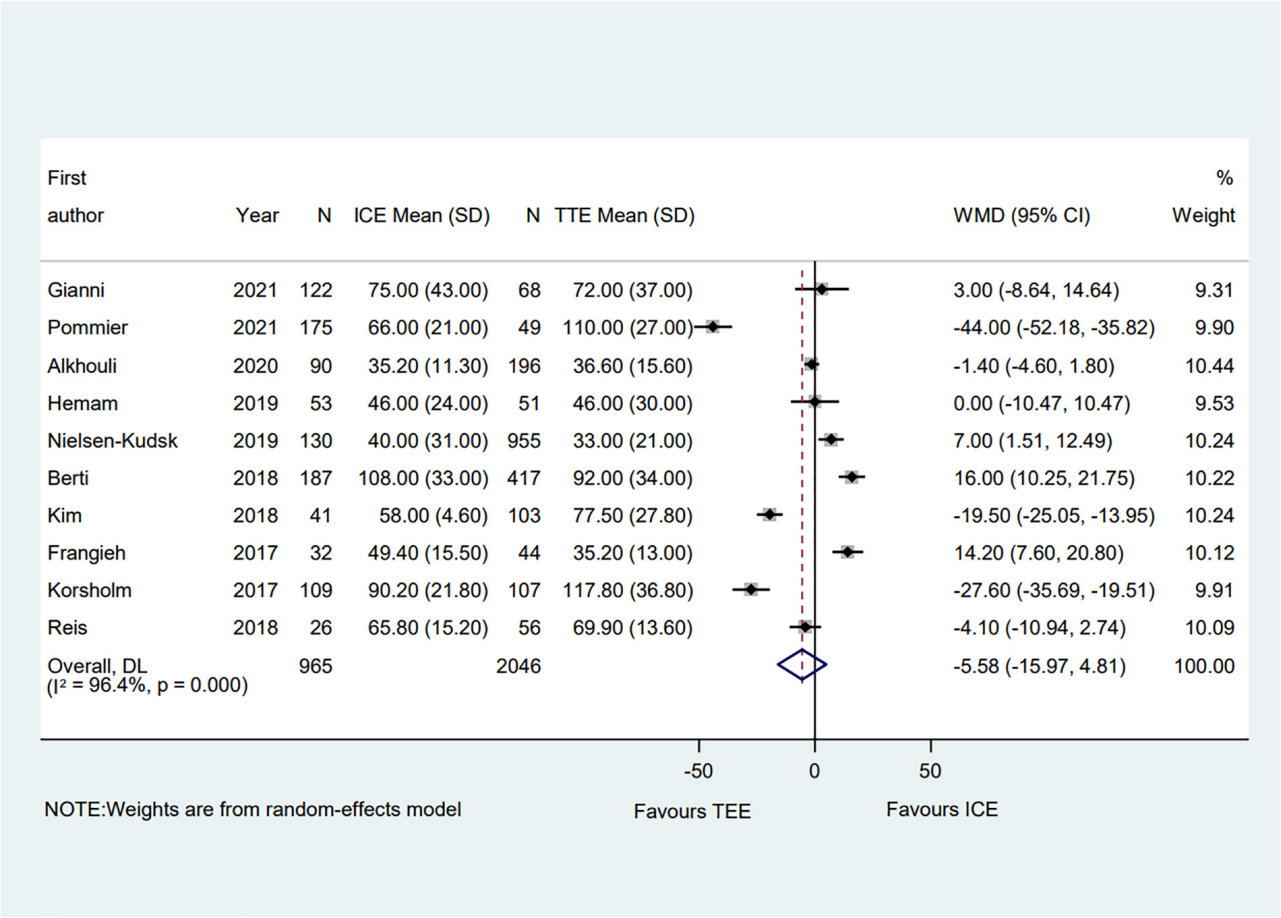
Forest plot of the total procedural time between ICE and TEE groups. Comparison of the rates of the total procedural time between ICE and TEE roups. ICE, intracardiac echocardiography; TEE, transesophageal echocardiography; WMD, weighted mean difference; CI, confidence interval.

A total of ten clinical studies provided the total procedural time, and the data on the total procedural time was similar between groups (WMD = −5.58; 95% CI: −15.97, 4.81; *P* = 0.29) (6–15). Significant heterogeneity was observed (*I*^2^ = 96.4%) (Figure 5). Subgroup analysis was performed with a total of seven subgroup factors for total procedure time, and the results are displayed in Supplementary Table S2. Interestingly, in the PAF proportion ≥50% subgroup, the procedural time in the TEE group was shorter than in the ICE group (WMD = 14.20; 95% CI: 7.6, 20.8; *P* = 0.000). Meanwhile, compared with the TEE group, the ICE group was associated with shorter procedural time in the muti-seal mechanism devices subgroup (WMD = −31.56; 95% CI: −55.57, −7.5; *P* = 0.010; *I*^2^ = 95.8%). Sensitivity analysis showed that no significant change, ranging from −7.80 (95% CI: −18.72, 3.11) to −1.35 (95% CI: −10.13, 7.44), was detected in the overall combined proportion. Moreover, no publication bias was shown in Egger's test (*P* = 0.535).

#### 3.3.2. Contrast volume

**Figure 6:**
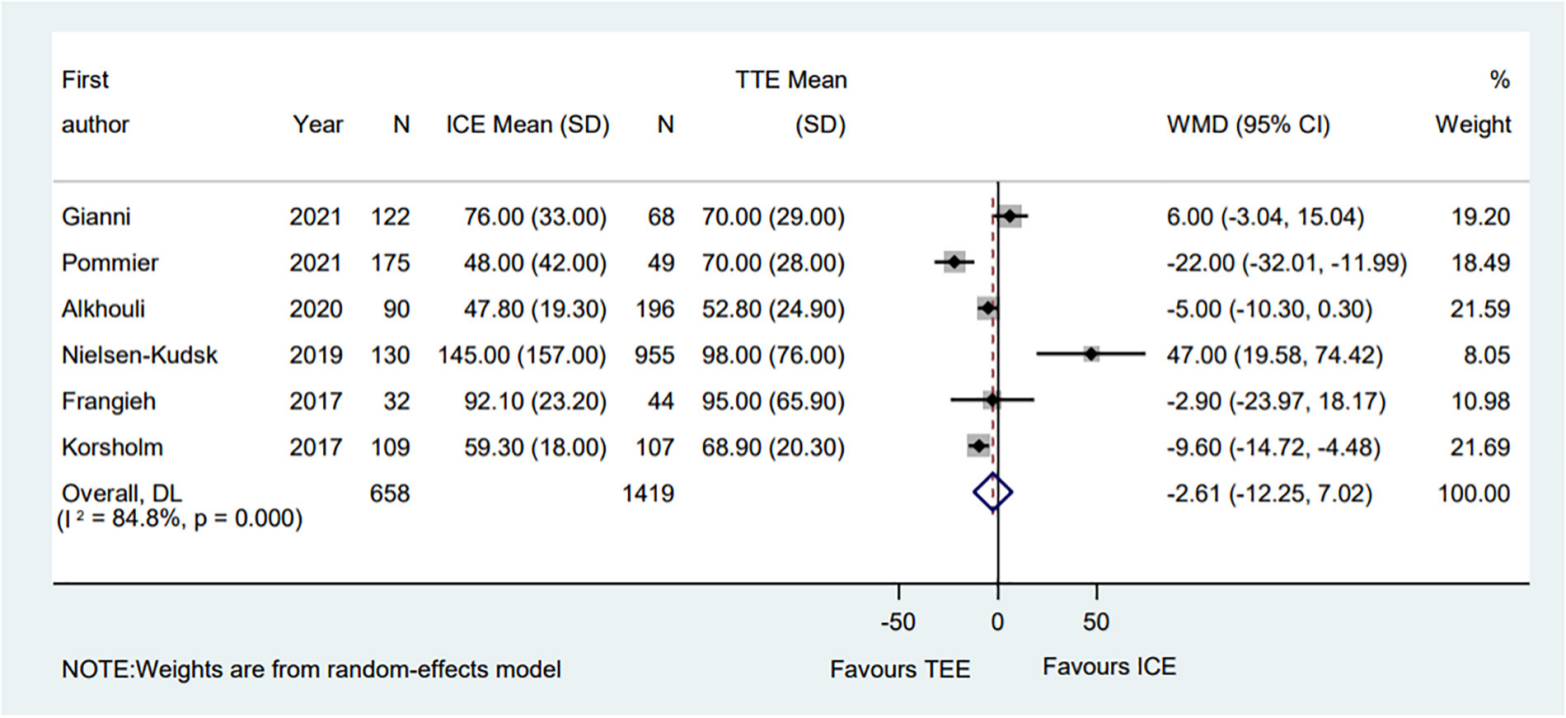
Forest plot of the contrast volume between ICE and TEE groups. Comparison of the rates of the contrast volume between ICE and TEE groups. ICE, intracardiac echocardiography; TEE, transesophageal echocardiography; WMD, weighted mean difference; CI, confidence interval.

A total of six eligible studies reported the contrast volume (6–8, 10, 13, 14). The pooled results indicated that compared with the TEE procedure, the ICE procedure showed no significant difference (WMD = −2.61; 95% CI: −12.25, 7.02; *P* = 0.595; *I*^2^ = 84.80%) (Figure 6). The subgroup analysis showed that in the PAF proportion <50% subgroup, the ICE group's contrast volume was significantly decreased compared with the TEE group (WMD = −15.02; 95% CI: −27.08, −2.97; *P* = 0.015; *I*^2^ = 78.60%). Moreover, in the hypertension proportion <90% subgroup, the contrast volume in the ICE group was much lower than that in the TEE group (WMD = −12.95; 95% CI: −22.83, −3.07; *P* = 0.010; *I*^2^ = 62.90%). Meanwhile, the ICE group was associated with less contrast volume than the TEE group in the muti-seal mechanism devices subgroup (WMD = −22.00; 95% CI: −32.01, −11.99; *P* = 0.000). Interestingly, in the muti-centered subgroup, ICE-guided LAAO required a greater amount of contrast volume than TEE-guided LAAO (WMD = 47.00; 95% CI: 19.59, 74.42; *P* = 0.001) (Supplementary Table S3).

Meanwhile, sensitivity analysis showed that no single study dominated the combined proportion and heterogeneity, ranging from −4.81 (95% CI: −15.29, 5.66) to 1.26 (95% CI: −8.55, 11.06). Moreover, Egger's test was performed and result showed no publication bias (*P* = 0.371), which suggested that the results were robust.

#### 3.3.3. Fluoroscopic time

**Figure 7:**
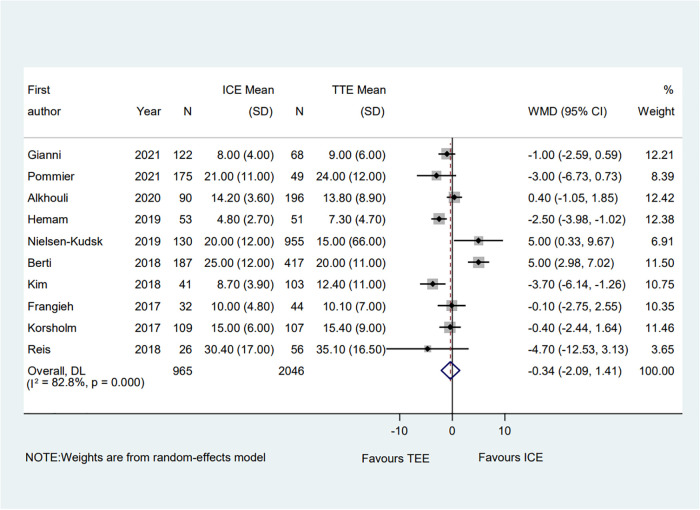
Forest plot of the fluoroscopic time between ICE and TEE groups. Comparison of the rates of the fluoroscopic time between ICE and TEE groups. ICE, intracardiac echocardiography; TEE, transesophageal echocardiography; WMD, weighted mean difference; CI, confidence interval.

A total of ten eligible studies reported the fluoroscopic time and the pooled result showed that the fluoroscopic time guided by ICE was significantly equivalent to that guided by TEE (WMD = −0.34; 95% CI: −2.09, 1.41; *P* = 0.705; *I*^2^ = 82.80%) (Figure 7) (6–15). Subgroup analysis was performed with a total of seven subgroup factors for the fluoroscopic time, and the results were displayed in Supplementary Table S4. Compared with the TEE group, the fluoroscopic time in the ICE group was much shorter in the hypertension proportion <90% subgroup (WMD = −1.49; 95% CI: −2.87, −0.10; *P *= 0.035; *I*^2^ = 33.50%) as well as the muti-seal mechanism devices subgroup (WMD = −3.49; 95% CI: −5.53, −1.45; *P *= 0.001; *I*^2^ = 0.00%). No significant change was detected in the overall combined proportion by sensitivity analysis, ranging from −0.72 (95% CI: −2.48, 1.03) to 0.07 (95% CI: −1.74, 1.87). Moreover, no publication bias was shown in Egger's test (*P* = 0.941).

#### 3.3.4. Pooled safety outcomes

**Figure 8:**
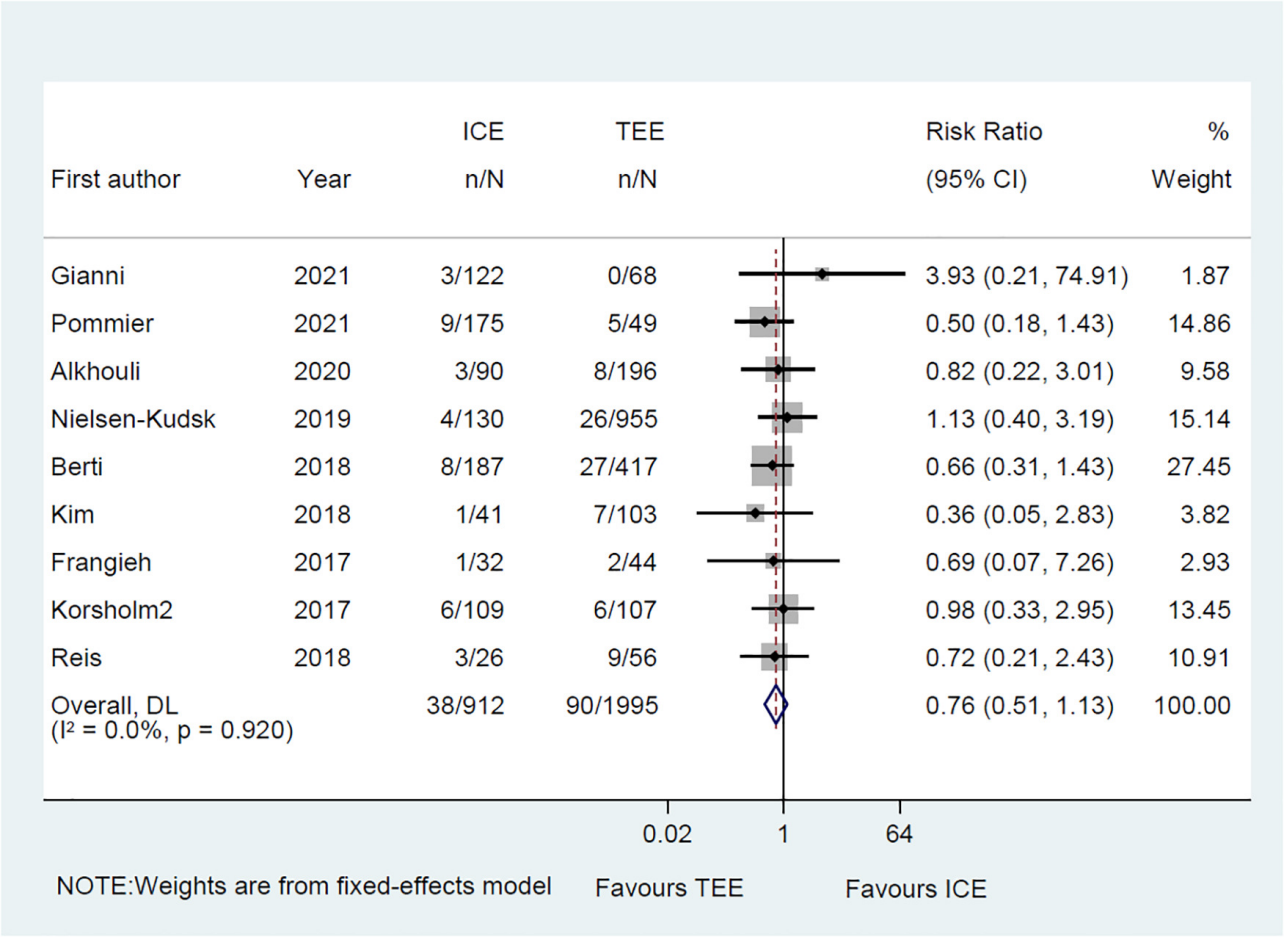
Forest plot of the preprocedural complications between ICE and TEE groups. Comparison of the rates of the preprocedural complications between ICE and TEE groups. ICE, intracardiac echocardiography; TEE, transesophageal echocardiography; RR, risk ratio; CI, confidence interval.

**Figure 9:**
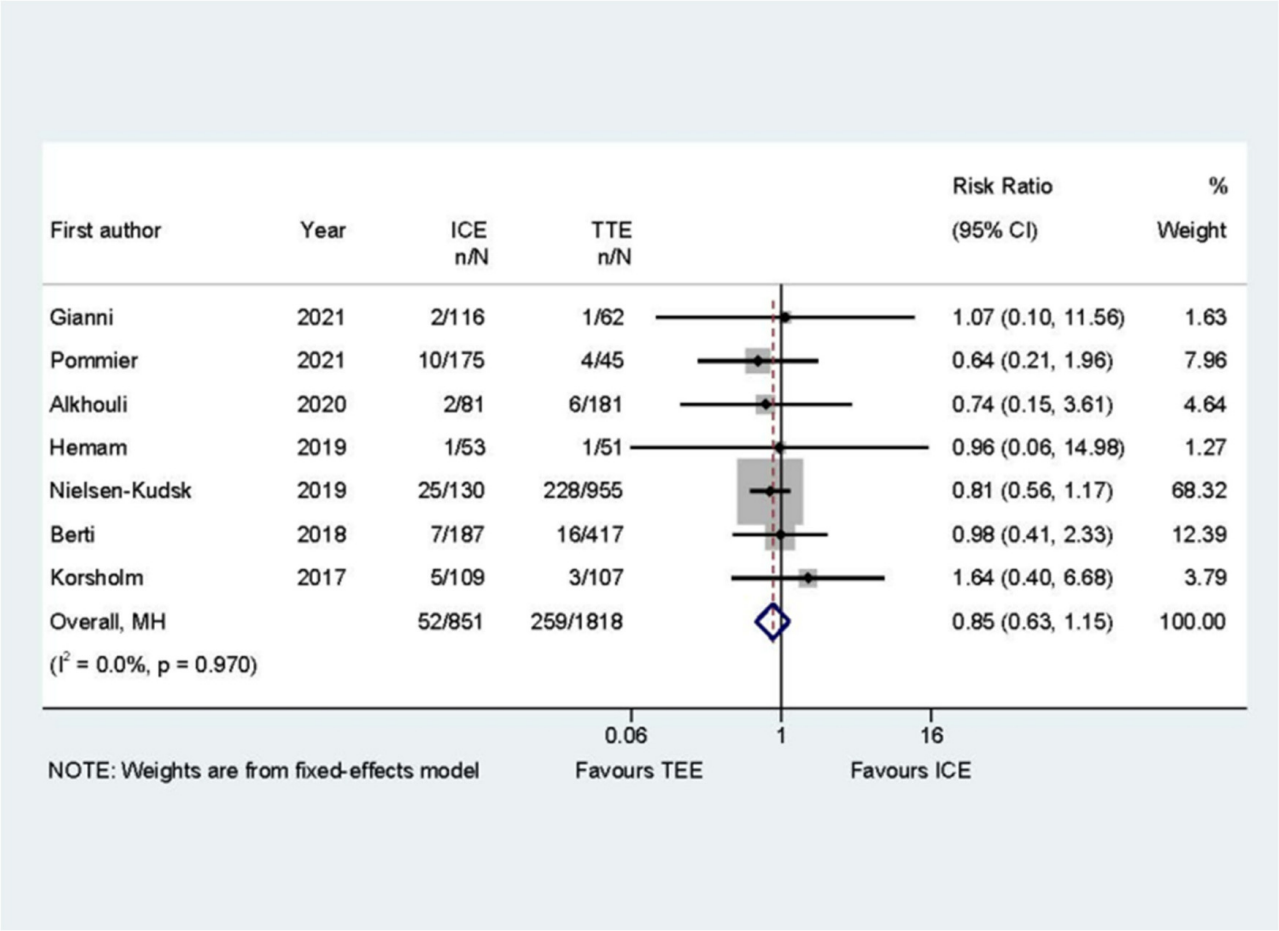
Forest plot of the long-term adverse events between ICE and TEE groups. Comparison of the rates of the long-term adverse events between ICE and TEE groups. ICE, intracardiac echocardiography; TEE, transesophageal echocardiography; RR, risk ratio; CI, confidence interval.

Common perioperative complications include cardiac effusion, cardiac tamponade, device migration, device thrombus, stroke/TIA, bleeding, hematoma, renal complications, cardiac arrest, and death. The data on procedural complications was available in nine clinical studies (6–8, 10–15). Complications from each eligible study were listed independently in Supplementary Tables S5, S6. The rate of procedural complications in ICE group was similar with that of TEE group (RR = 0.82; 95% CI: 0.58, 1.16; *P* = 0.261; *I*^2^ = 23.50%) (Figure 8). Sensitivity analysis was performed and the results showed no significant change in the overall combined proportion, ranging from 0.70 (95% CI: 0.45, 1.10) to 0.87 (95% CI: 0.61, 1.25). Egger's test also showed no publication bias (*P* = 0.696). Meanwhile, seven clinical studies were followed up and reported long-term adverse events (6–11, 14). In terms of long-term adverse events, the ICE group showed a similar result to TEE group (RR = 0.86; 95% CI: 0.64, 1.16; *P* = 0.329; *I*^2^ = 41.10%) (Figure 9).

## 4. Discussion

Among twenty enrolled published original articles, a total of 3,610 patients (including 1,564 patients for ICE and 2,046 patients for TEE) were evaluated. Compared with previous meta-analysis, we included recent publications and single-arm studies. Meanwhile we performed subgroup analysis for each endpoint event. Our main findings were as follows. Compared with TEE group, (1) ICE group showed comparable efficacy and safety outcomes for LAAO, including the acute procedural success rate, total procedure time, contrast volume, the fluoroscopic time, and safety outcomes; (2) ICE group might reduce the use of contrast agent and fluoroscopic time in the hypertension proportion <90 subgroup; (3) ICE group might be associated with lower total procedure time, contrast volume, and the fluoroscopic time in device type subgroup with multi-seal mechanism; (4) The total procedure time might be longer in PAF proportion >50 subgroup while the contrast use might be less in PAF proportion ≤50 subgroup for ICE group; (5) ICE group might be related in an increased use of contrast in multi-center subgroup.

AF is an important pathogenesis of ischemic stroke, with approximately 5% of stroke patients being associated with AF each year, ultimately resulting in high rates of mortality and morbidity (3). LAAO has been demonstrated to be an alternative to prevent stroke in AF patients, particularly for individuals who are intolerant to oral anticoagulants. Intraoperative imaging is a crucial factor for LAAO. While TEE is currently the mainstream method, ICE is increasingly being used as an alternative to TEE.

In this meta-analysis, we compared the acute procedural success between the TEE and ICE groups. Similar with previous studies (26–28), we found no significant difference between the two groups. We then conducted a subgroup analysis to further compare the advantages and disadvantages of the two groups. The result showed that, regardless of the subgroup, there was no significant difference in acute procedural success rate. TEE is the gold standard imaging method for LAAO, providing clear images of the right atrium, left atrium, atrial septum, and left atrial appendage anatomy for LAAO. However, TEE-guided LAAO has some disadvantages, such as increased pain with local or conscious anesthesia, prolonged procedure time and hospitalization burden with general anesthesia, aggravated risk of possible esophageal injury under “one-stop” ablation, and high dependence on a dedicated echocardiography operator. To explore the safety of ICE and TEE, we recorded both the preprocedural complications and the long-term complications. For the short-term adverse events, the results showed that ICE was not inferior to TEE in guiding LAA occlusion procedures in terms of peri-procedural complications. Additionally, the long-term adverse events were comparable between groups, indicating that ICE had a reliable performance on safety.

Hypertension is one of the common comorbidities and modifiable risk factors in cardiovascular diseases, which could lead to the enlargement of left atria diameter, promotion of atrial fibrosis, and impairment of the endothelial function, ultimately causing the initiation and progression of AF and related stroke (29). However, few studies reported the role of hypertension on the procedure of LAAO for AF. Our subgroup results showed that ICE group might reduce the use of contrast agent and fluoroscopic time in the hypertension proportion <90 subgroup in comparison with the TEE group, suggesting that the lower proportion of hypertension may be associated with the more benefit for AF patients with LAAO procedure. This result might provide a basis for a randomized control trial to further evaluate the role of hypertension on the use of contrast agent and the fluoroscopic time between ICE-guided and TEE-guided LAAO.

At present, multiple types of devices for LAAO were applied in clinical procedure, mainly including single-seal mechanism device, dual-seal mechanism devices, and both mechanism device (30). Accumulated studies had revealed that selective application of the device type for LAAO might showed a similarly clinical outcomes based on the specific morphologies of LAA (31). Interestingly, ICE-guided LAAO might be associated with lower total procedure time, contrast volume, and the fluoroscopic time in device type subgroup with multi-seal mechanism. We could make a reasonable speculation that the application of multi-seal mechanism devices is associated with the mastery of the ICE-guided LAAO procedure by operators. Whereas, more studies should be performed to demonstrate this result.

Studies on the impact of AF type during LAAO procedure are emerging. A recent lesson from the prospective Left Atrial Appendage Occluder Registry Germany (LAARGE) had suggested that the procedure time and fluoroscopy time were longer for LAAO procedure in PAF patients than non-PAF patients, which might be significantly related in the challenge of LAA movement due to the higher rate of sinus rhythm in PAF patients during LAAO procedure (32). Similarly, our subgroup also indicated that the total procedure time in ICE-guided LAAO might be longer in PAF proportion >50 subgroup. In addition, the contrast use might be less in PAF proportion ≤50 subgroup for ICE-guided LAAO group, potentially suggesting that ICE-guided LAAO might reduce the contrast use for non-PAF patients.

Additionally, our subgroup results suggested that ICE-guided LAAO might be associated with an increased use of contrast in multi-center subgroup, which indicated that ICE-guided LAAO showed unsatisfied performance on the contrast use in multi-center subgroup in comparison with single-center subgroup. This might be explained by the multiple possibilities, including center heterogeneity, team quality heterogeneity, and relatively rigid procedure protocol rarely with decision-making strategy in multi-center study. Moreover, only one multi-center study (10) reported the contrast use for subgroup analysis, which might cause potential bias due to the limited sample size. Therefore, more prospective studies are needed to further demonstrate our results.

Also, a total of two studies compared the cost of hospitalization between ICE group and TEE group (8, 9), which showed that the global charges were similar between the ICE-guided LAAO and TEE-guided LAAO in American centers. Whereas, in other medical centers, the hospital charges of ICE-guided LAAO might be higher in comparison with TEE-guided LAAO due to the higher cost of ICE catheter (33). Also, local medical team experience and environment would play an important role on the determination of the appropriate imaging modality to be implemented. Therefore, more prospective, randomized studies will probably clarify the comparison of ICE and TEE for guiding the LAAO procedure, especially in terms of efficacy, safety, and hospital charges.

## 5. Limitations

Our study has several limitations. First, the studies included in this meta-analysis were nonrandomized and observational in design, which might lead to potential selection bias. Second, the sample size included in the study is small which may affect the stability of the result indicators, reduce the efficiency of the test, and introduce potential research bias. Third, different studies were followed with different tests, which may have affected the follow-up results. In addition, clinical studies lacked a uniform definition of procedural success and procedure-related complications. Therefore, a prospective, randomized study is needed to clarify the clinical outcomes of LAAO with the comparison of ICE vs. TEE monitoring.

## 6. Conclusions

Our results demonstrate that ICE may have comparable efficacy and safety compared to TEE for LAAO.

## Data Availability

The data that support the findings of this study are available from the corresponding author upon reasonable request.
