# Correction: A comparable efficacy and safety between intracardiac echocardiography and transesophageal echocardiography for percutaneous left atrial appendage occlusion

**DOI:** 10.3389/fcvm.2026.1816654

**Published:** 2026-05-15

**Authors:** Zhi-Yuan Zhang, Feng Li, Jie Zhang, Lei Zhang, Huan-Huan Liu, Ning Zhao, Fan Yang, Qi Kong, Yi-Ting Zhou, Ling-Ling Qian, Ru-Xing Wang

**Affiliations:** Department of Cardiology, Wuxi People's Hospital Affiliated to Nanjing Medical University, Wuxi, China

**Keywords:** atrial fibrillation, intracardiac echocardiography, transesophageal echocardiography, left atrial appendage closure, implantable devices, cardiac mapping

The published versions of Figure 9 and Supplementary Table 6 contained an error in the reporting of long-term adverse events. The correct methodology was as follows: long-term adverse events were derived by retaining the relevant follow-up events from the original data after excluding procedure- or device-related serious adverse events (SAEs) and short-term adverse events, and by including cases with leaks ≥5 mm.

The corrected long-term adverse events are:

**ICE group:** Death (8), Cerebrovascular disease (6), Major bleeding (10), Renal complications (1).

**TEE group:** Death (79), Cerebrovascular disease (30), Major bleeding (96), Device-related outcomes (2), Renal complications (21).

A miscalculation was identified in the long-term adverse events analysis. Specifically, the number of major bleeding cases was 96 in the TEE group and 10 in the ICE group. As a result, the corrected total number of complications was 4 in the ICE group and 26 in the TEE group, and the total number of complications recorded at follow-up was 25 in the ICE group and 228 in the TEE group.

This error does not affect the study's conclusions or the statistical significance of the reported outcomes.

The correct versions of Figure 9 and Supplementary Table 6 are provided below:

**Figure 9:**
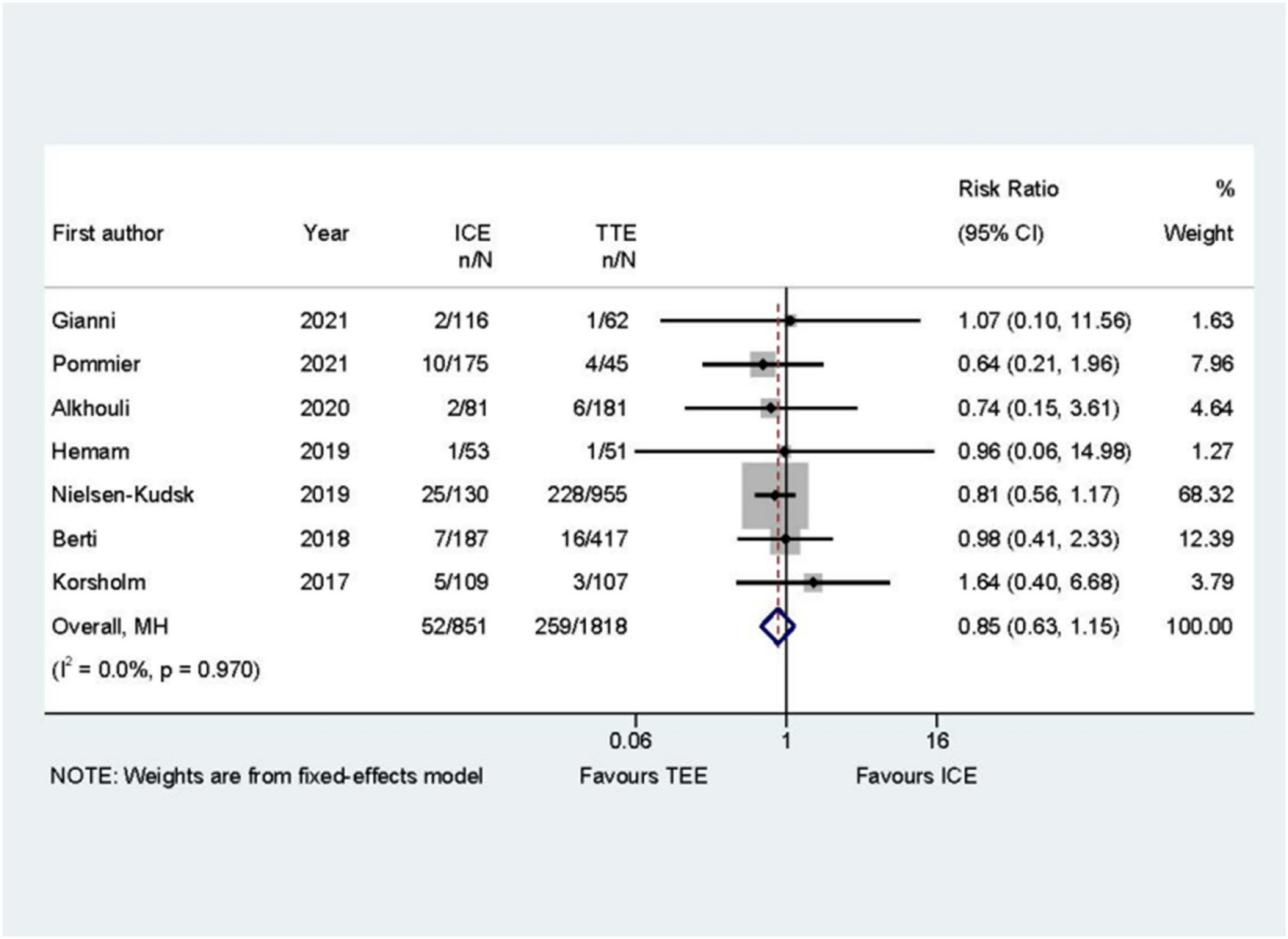
Forest plot of the long-term adverse events between ICE and TEE groups. Comparison of the rates of long-term adverse events between ICE and TEE groups. ICE: intracardiac echocardiography; TEE: transesophageal echocardiography; RR: risk ratio; CI: confidence interval.

**Supplementary Table 6:** Follow up complications between ICE group and TEE group.

Study	Year	Follow up (months)	ICE	TEE
Gianni	2021	2	Device-related outcomes (2)	Device-related outcomes (1)
Pommier	2021	1	Device-related outcomes (10)	Device-related outcomes (5)
Alkhouli	2020	1.5	Device-related outcomes (2)	Device-related outcomes (6)
Hemam	2019	4	Device-related outcomes (1)	Device-related outcomes (1)
Nielsen-Kudsk	2019	12	Death (8), Cerebrovascular disease (6), Major bleeding (10), Renal complications (1)	Death (79), Cerebrovascular disease (30), Major bleeding (96), Device-related outcomes (2), Renal complications (21)
Berti	2018	15	Cerebrovascular diseases (7)	Cerebrovascular diseases (16)
Kim	2018	25.6	–	–
Frangieh	2017	0	–	–
Korsholm2	2017	1.7	Cerebrovascular diseases (1), Device-related outcomes (1), Major bleeding (3)	Cerebrovascular diseases (2), Device-related outcomes (1)
Reis	2018	23	Device-related outcomes (1), Major bleeding (3), Cerebrovascular diseases (1), Death (4)	
Dallan	2022	1.5	Device-related outcomes (1)	
Turagam1	2022	12	Death (2)	
Chen	2022	12	Death (1), Device-related outcomes (2), Major bleeding (1)	
Turagam2	2021	1.5	Death (1), Major bleeding (2)	
Filby	2021	1.5	–	
Korsholm1	2020	1.7	Death (1), Cerebrovascular diseases (1)	
Khalili	2019	0	–	–
Matsuo	2016	1.5	Device-related outcomes (3)	
Masson	2015	2	Death (2)	
Berti	2014	0	–	–

NOTE: Cerebrovascular Diseases: Ischemic stroke, TIA and Cerebral hemorrhage; Device-related outcomes: Device thrombus, Device migration and ≥5mm peri-device flow; Major bleeding: Cardiac effusion, Cardiac tamponade and Major bleeding event; Renal complications: Acute renal failure, Chronic renal failure, Renal insufficiency, Acute kidney injury and Cardiorenal syndrome.

The original version of this article has been updated.

