# Supplementary material for: A comparable efficacy and safety between intracardiac echocardiography and transesophageal echocardiography for percutaneous left atrial appendage occlusion
Source: Front Cardiovasc Med. 2023 May 24;10:1194771. doi: 10.3389/fcvm.2023.1194771 (PMC10244765; doi:10.3389/fcvm.2023.1194771)
Supplement: Supplementary file 1 [file Table3.docx]

**Supplementary Table1** Quality assessment of eligible studies according to the Newcastle-Ottawa Quality Assessment Scale

| First author | Year | Selection | | | | Comparability | Outcome | | | Total stars |
| --- | --- | --- | --- | --- | --- | --- | --- | --- | --- | --- |
|  |  | Representative of the exposed cohort | Selection of the nonexposed cohort | Ascertainment of exposure | Demonstration that outcome of interest was not present at start of study | Comparability of cohorts on the basis  of the design or analysis | Assessment of outcome | Was follow-up long enough for outcomes to occur | Adequacy of follow-up of cohorts |  |
| Gianni | 2021 | **☆** | **☆** | **☆** | **☆** | **☆** | **☆** | **☆** | **☆** | 8 |
| Pommier | 2021 | **☆** | **☆** | **☆** | **☆** | **☆** | **☆** | **☆** | **☆** | 8 |
| Alkhouli | 2020 | **☆** | **☆** | **☆** | **☆** | **☆** | **☆** | **☆** | **☆** | 8 |
| Hemam | 2019 | **☆** | **☆** | **☆** | **☆** | **☆** | **☆** | **☆** | **☆** | 8 |
| Nielsen-Kudsk | 2019 | **☆** | **☆** | **☆** | **☆** | **☆** | **☆** | **☆** | **☆** | 8 |
| Berti | 2018 | **☆** | **☆** | **☆** | **☆** | **☆** | **☆** | **☆** | **☆** | 8 |
| Kim | 2018 | **☆** | **☆** | **☆** | **☆** | **☆** | **☆** | **☆** | **☆** | 8 |
| Frangieh | 2017 | **☆** | **☆** | **☆** | **☆** | **☆** | **☆** |  |  | 6 |
| Korsholm2 | 2017 | **☆** | **☆** | **☆** | **☆** | **☆** | **☆** | **☆** | **☆** | 8 |
| Reis | 2018 | **☆** | **☆** | **☆** | **☆** | **☆** | **☆** | **☆** | **☆** | 8 |

Quality assessment of the single-arm studies according to the Institute of Health Economics checklist

| First author | Year | Study objective | Study design | Study population | Intervention and cointervention | Outcome measures | Statistical analysis | Results and conclusions | Quality score |
| --- | --- | --- | --- | --- | --- | --- | --- | --- | --- |
| Dallan | 2022 | 1 | 2 | 3 | 2 | 3 | 1 | 5 | 17 |
| Turagam1 | 2022 | 1 | 2 | 3 | 2 | 3 | 1 | 5 | 17 |
| Chen | 2022 | 1 | 2 | 3 | 2 | 3 | 1 | 5 | 17 |
| Turagam2 | 2021 | 1 | 2 | 3 | 2 | 3 | 1 | 5 | 17 |
| Filby | 2021 | 1 | 2 | 3 | 2 | 3 | 1 | 5 | 17 |
| Korsholm1 | 2020 | 1 | 2 | 3 | 2 | 3 | 1 | 5 | 17 |
| Khalili | 2019 | 1 | 2 | 3 | 2 | 3 | 1 | 3 | 15 |
| Matsuo | 2016 | 1 | 2 | 3 | 2 | 3 | 1 | 5 | 17 |
| Masson | 2015 | 1 | 2 | 3 | 2 | 3 | 1 | 3 | 15 |
| Berti | 2014 | 1 | 3 | 3 | 2 | 3 | 1 | 5 | 18 |
