# Supplementary material for: A comparable efficacy and safety between intracardiac echocardiography and transesophageal echocardiography for percutaneous left atrial appendage occlusion
Source: Front Cardiovasc Med. 2023 May 24;10:1194771. doi: 10.3389/fcvm.2023.1194771 (PMC10244765; doi:10.3389/fcvm.2023.1194771)
Supplement: Supplementary file 2 [file Table4.docx]

**Supplementary Table 2.** Subgroup analysis of total procedure time between ICE group and TEE group

| Subgroup factors | Numbers of study | WMD (95%CI) | I^2^ (%) | *P* value | *P* for interaction |
| --- | --- | --- | --- | --- | --- |
| Study design |  |  |  |  | 0.344 |
| Multi-center | 4 | 0.88 (-15.84, 17.61) | 96.4 | 0.918 |  |
| Single-center | 6 | -9.94 (-24.90, 5.02) | 96.8 | 0.193 |  |
| ICE Sample size |  |  |  |  | 0.588 |
| >100 | 5 | -9.07 (-31,36, 13.22) | 97.9 | 0.425 |  |
| ≤100 | 5 | -2.28 (-12.52, 7.95) | 93.5 | 0.662 |  |
| Male proportion |  |  |  |  | 0.694 |
| <70 | 7 | -3.21 (-13.70, 7.28) | 95.2 | 0.548 |  |
| ≥70 | 2 | -5.75 (-71.88, 42.19) | 99.2 | 0.610 |  |
| Age cutoff |  |  |  |  | 0.800 |
| ≥75 | 5 | -4.30 (-20.3, 11.43) | 97.3 | 0.592 |  |
| <75 | 4 | -7.58 (-27.53, 12.38) | 96.5 | 0.457 |  |
| HT proportion |  |  |  |  | 0.810 |
| <90 | 4 | -14.35 (-42.06, 13.37) | 97.8 | 0.310 |  |
| ≥90 | 2 | -10.30 (-28.04, 7.43) | 96.7 | 0.255 |  |
| PAF proportion |  |  |  |  | 0.014 |
| >50 | 1 | 14.20 (7.60, 20.80) | - | 0.000 |  |
| ≤50 | 4 | -18.66 (-43.95, 6.63) | 98.2 | 0.148 |  |
| Devices type |  |  |  |  | 0.024 |
| Dual-seal mechanism | 3 | -1.35 (-23.92, 21.23) | 97.4 | 0.907 |  |
| Single-seal mechanism | 4 | 4.02 (-4.61, 12.65) | 82.9 | 0.361 |  |
| Muti-seal mechanism | 2 | -31.56 (-55.57, -7.55) | 95.8 | 0.010 |  |

Note: ICE: intracardiac echocardiography; TEE: transesophageal echocardiography; WMD: weighted mean difference; CI: confidence interval.
