# Supplementary material for: A comparable efficacy and safety between intracardiac echocardiography and transesophageal echocardiography for percutaneous left atrial appendage occlusion
Source: Front Cardiovasc Med. 2023 May 24;10:1194771. doi: 10.3389/fcvm.2023.1194771 (PMC10244765; doi:10.3389/fcvm.2023.1194771)
Supplement: Supplementary file 3 [file Table5.docx]

**Supplementary Table 3.** Subgroup analysis of Contrast volume between ICE group and TEE group

| Subgroup factors | Numbers of study | WMD (95%CI) | I^2^ (%) | *P* value | *P* for interaction |
| --- | --- | --- | --- | --- | --- |
| Study design |  |  |  |  | 0.000 |
| Multi-center | 1 | 47.00(19.58, 74.42) | - | 0.001 |  |
| Single-center | 5 | -7.01(-14.76, 0.75) | 78.1 | 0.076 |  |
| ICE Sample size |  |  |  |  | 0.536 |
| >100 | 4 | 0.48(-15.67, 16.64) | 90.8 | 0.953 |  |
| ≤100 | 2 | -4.88(-10.01, 0.26) | 0.00 | 0.063 |  |
| Male proportion |  |  |  |  | 0.117 |
| <70 | 4 | 2.17(-8.78,13.12) | 86.7 | 0.697 |  |
| ≥70 | 3 | -14.79(-32.94,3.36) | 61.2 | 0.110 |  |
| Age cutoff |  |  |  |  | 0.750 |
| ≥75 | 3 | 1.60(-20.59, 23.79) | 91.7 | 0.887 |  |
| <75 | 3 | -2.50(-14.51, 9.51) | 77.1 | 0.683 |  |
| HT proportion |  |  |  |  | 0.164 |
| <90 | 3 | -12.95(-22.83, -3.07) | 62.9 | 0.010 |  |
| ≥90 | 1 | -5.00(-10.30, 0.30) | - | 0.064 |  |
| PAF proportion |  |  |  |  | 0.328 |
| >50 | 1 | -2.90(-23.97, 18.17) | - | 0.787 |  |
| ≤50 | 2 | -15.02(-27.08, -2.97) | 78.6 | 0.015 |  |
| Devices type |  |  |  |  | 0.004 |
| Dual-seal mechanism | 2 | 17.03(-38.34, 72.40) | 93.7 | 0.860 |  |
| Single-seal mechanism | 3 | -0.74(-8.49, 7.46) | 52.8 | 0.547 |  |
| Muti-seal mechanism | 1 | -22.00(-32.01, -11.99) | - | 0.000 |  |

Note: ICE: intracardiac echocardiography; TEE: transesophageal echocardiography; WMD: weighted mean difference; CI: confidence interval.
