# Supplementary material for: A comparable efficacy and safety between intracardiac echocardiography and transesophageal echocardiography for percutaneous left atrial appendage occlusion
Source: Front Cardiovasc Med. 2023 May 24;10:1194771. doi: 10.3389/fcvm.2023.1194771 (PMC10244765; doi:10.3389/fcvm.2023.1194771)
Supplement: Supplementary file 4 [file Table6.docx]

**Supplementary table 4.** Subgroup analysis of fluoroscopic time between ICE group and TEE group

| **Subgroup Factors** | **Numbers of Study** | **WMD (95% CI)** | | **I^2^ (%)** | ***P* value** | ***P* for interaction** |
| --- | --- | --- | --- | --- | --- | --- |
| Study design |  |  |  |  |  | 0.608 |
| Multi-center | 4 | 0.763(-3.80,5.32) | | 93.7 | 0.036 |  |
| Single-center | 6 | -0.453(-1.32,0.41) | | 0.0 | 0.305 |  |
| ICE Sample size |  |  |  |  |  | 0.145 |
| >100 | 5 | 1.02(-1.96,4.0) | | 86.6 | 0.503 |  |
| ≤100 | 5 | -1.59(-3.44,0.26) | | 69.3 | 0.092 |  |
| Male proportion |  |  |  |  |  | 0.437 |
| <70 | 7 | 0.14(-1.96,2.25) | | 87.8 | 0.894 |  |
| ≥70 | 2 | -1.24(-4.02,1.54) | | 35.0 | 0.381 |  |
| Age cutoff |  |  |  |  |  | 0.232 |
| ≥75 | 5 | 0.85(-2.28,3.98) | | 90.4 | 0.594 |  |
| <75 | 4 | -1.25(-2.67,0.17) | | 43.9 | 0.086 |  |
| HT proportion |  |  |  |  |  | 0.984 |
| <90 | 4 | -1.49(-2.87,-0.10) | | 33.5 | 0.035 |  |
| ≥90 | 2 | -1.39(-5.54,2.84) | | 87.5 | 0.455 |  |
| PAF proportion |  |  |  |  |  | 0.899 |
| >50 | 1 | -0.10(-2.75,2.55) | | - | 0.941 |  |
| ≤50 | 4 | -0.42(-4.54,3.71) | | 91.3 | 0.843 |  |
| Devices type |  |  |  |  |  | 0.011 |
| Dual-seal mechanism | 3 | 3.02(-1.08,7.12) | | 86.4 | 0.149 |  |
| Single-seal mechanism | 4 | -0.87(-2.27,0.52) | | 62.2 | 0.218 |  |
| Muti-seal mechanism | 2 | -3.49(-5.53,1.45) | | 0.0 | 0.001 |  |

Note: ICE: intracardiac echocardiography; TEE: transesophageal echocardiography; WMD: weighted mean difference; CI: confidence interval.
