# Supplementary material for: A comparable efficacy and safety between intracardiac echocardiography and transesophageal echocardiography for percutaneous left atrial appendage occlusion
Source: Front Cardiovasc Med. 2023 May 24;10:1194771. doi: 10.3389/fcvm.2023.1194771 (PMC10244765; doi:10.3389/fcvm.2023.1194771)
Supplement: Supplementary file 5 [file Table7.docx]

**Supplementary** **Table5**. Preprocedural complications between ICE group and TEE group.

| Study | Year | ICE | TEE |
| --- | --- | --- | --- |
| Gianni | 2021 | Major bleeding (3) | - |
| Pommier | 2021 | Major bleeding (4), Vascular complications (1), Device-related outcomes (3), Cerebrovascular diseases (1) | Major bleeding (4), Device-related outcomes (1) |
| Alkhouli | 2020 | Major bleeding (1), Vascular complications (1), Cerebrovascular diseases (1) | Major bleeding (4), Device-related outcomes (1), Death (1), Vascular complications (1) |
| Hemam | 2019 | - | - |
| Nielsen-Kudsk | 2019 | Vascular complications (1), Major bleeding (3) | Vascular complications (14), Major bleeding (12), |
| Berti | 2018 | Device-related outcomes (1), Cerebrovascular diseases (1), Major bleeding (6) | Device-related outcomes (1), Cerebrovascular diseases (2), Cardiac effusion or tamponade (8), Major bleeding (16) |
| Kim | 2018 | Major bleeding (1) | Device-related outcomes (3), Major bleeding (3), vascular complication (1) |
| Frangieh | 2017 | Major bleeding (1) | Death (1), Major bleeding (1) |
| Korsholm2 | 2017 | Major bleeding (2), Vascular complication (4) | Device-related outcomes (1), Cerebrovascular diseases (2), Major bleeding (2), Vascular complication (1) |
| Reis | 2018 | Major bleeding (3) | Device-related outcomes (5), Major bleeding (4) |
| Dallan | 2022 | Cardiac arrest (1), Major bleeding (7), vascular complication (1) |  |
| Turagam^1^ | 2022 | - |  |
| Chen | 2022 | Major bleeding (2) |  |
| Turagam^2^ | 2021 | - |  |
| Filby | 2021 | Cardiac arrest (1), Major bleeding (1) |  |
| Korsholm1 | 2020 | Major bleeding (5) |  |
| Khalili | 2019 | - | - |
| Matsuo | 2016 | Cerebrovascular diseases (1), Vascular complications (3) |  |
| Masson | 2015 | Cardiac arrest (1), Major bleeding (2) |  |
| Berti | 2014 | Device-related outcomes (1), Cerebrovascular diseases (1), Major bleeding (2), Vascular complication (2) |  |

Note: Cerebrovascular diseases: Ischemic stroke, TIA, cerebral hemorrhage; Device-related outcomes: Device thrombus, Device migration, ≥5mm peri-device flow; Major bleeding: Cardiac effusion, Cardiac tamponade, Major bleeding event.
