# Supplementary material for: A comparable efficacy and safety between intracardiac echocardiography and transesophageal echocardiography for percutaneous left atrial appendage occlusion
Source: Front Cardiovasc Med. 2023 May 24;10:1194771. doi: 10.3389/fcvm.2023.1194771 (PMC10244765; doi:10.3389/fcvm.2023.1194771)
Supplement: Supplementary file 6 [file Table8.docx]

**Supplementary** **Table 6**. Follow up complications between ICE group and TEE group.

| Study | Year | Follow up(months) | ICE | TEE |
| --- | --- | --- | --- | --- |
| Gianni | 2021 | 2 | Device-related outcomes (2) | Device-related outcomes (1) |
| Pommier | 2021 | 1 | Device-related outcomes (10) | Device-related outcomes (5) |
| Alkhouli | 2020 | 1.5 | Device-related outcomes (2) | Device-related outcomes (6) |
| Hemam | 2019 | 4 | Device-related outcomes (1) | Device-related outcomes (1) |
| Nielsen-Kudsk | 2019 | 12 | Death (8), Cerebrovascular disease (6), Major bleeding (10), Renal complications (1) | Death (79), Cerebrovascular disease (30), Major bleeding (96), Device-related outcomes (2), Renal complications (21) |
| Berti | 2018 | 15 | Cerebrovascular diseases (7) | Cerebrovascular diseases (16) |
| Kim | 2018 | 25.6 | - | - |
| Frangieh | 2017 | 0 | - | - |
| Korsholm2 | 2017 | 1.7 | Cerebrovascular diseases (1), Device-related outcomes (1), Major bleeding (3) | Cerebrovascular diseases (2), Device-related outcomes (1) |
| Reis | 2018 | 23 | Device-related outcomes (1), Major bleeding (3), Cerebrovascular diseases (1), Death (4) | |
| Dallan | 2022 | 1.5 | Device-related outcomes (1) |  |
| Turagam1 | 2022 | 12 | Death (2) |  |
| Chen | 2022 | 12 | Death (1), Device-related outcomes (2), Major bleeding (1) |  |
| Turagam2 | 2021 | 1.5 | Death (1), Major bleeding (2) |  |
| Filby | 2021 | 1.5 | - |  |
| Korsholm1 | 2020 | 1.7 | Death (1), Cerebrovascular diseases (1) |  |
| Khalili | 2019 | 0 | - | - |
| Matsuo | 2016 | 1.5 | Device-related outcomes (3) |  |
| Masson | 2015 | 2 | Death (2) |  |
| Berti | 2014 | 0 | - | - |

NOTE: Cerebrovascular Diseases: Ischemic stroke, TIA and Cerebral hemorrhage; Device-related outcomes: Device thrombus, Device migration and ≥5mm peri-device flow; Major bleeding: Cardiac effusion, Cardiac tamponade and Major bleeding event; Renal complications: Acute renal failure, Chronic renal failure, Renal insufficiency, Acute kidney injury and Cardiorenal syndrome.
